# The effect of storage at ambient temperature on the feline fecal microbiota

**DOI:** 10.1186/s12917-017-1188-z

**Published:** 2017-08-18

**Authors:** Moran Tal, Adronie Verbrugghe, Diego E. Gomez, Charlotte Chau, J. Scott Weese

**Affiliations:** 1 Department of Clinical Studies, Ontario Veterinary College, University of Guelph, Guelph, ON N1G 2W1 Canada; 2 Department of Pathobiology, Ontario Veterinary College, University of Guelph, Guelph, ON N1G 2W1 Canada; 3 College of Veterinary Medicine, University of Florida, Gainesville, Florida USA

**Keywords:** Gastrointestinal, Fecal, Microbiota, Storage, Temperature, Ambient

## Abstract

**Background:**

Feline fecal microbiota analyses can potentially be impacted by a variety of factors such as sample preparation, sequencing method and bioinformatics analyses. Another potential influence is changes in the microbiota from storage of samples prior to processing. This study examined the effect of ambient temperature exposure on the feline fecal microbiota composition.

Fecal samples were collected from 12 healthy cats, within 15 min after defecation. Samples were aliquoted and the first aliquot was frozen at −80 °C within 1 hour of defecation. Remaining aliquots were maintained at ambient temperature (20 to 23 °C) and frozen at −80 °C at 6, 12, 24, 36, 48, 72 and 96 h after collection. DNA was extracted from all aliquots, and polymerase chain reaction (PCR). The PCR products were sequenced with next-generation sequencing (Illumina MiSeq).

**Results:**

No significant differences were observed in alpha and beta biodiversity indexes, as well as relative abundance of different taxa over time (*P* > 0.05 for all tests between time points). Principal coordinate analyses demonstrated that samples cluster mainly by cat, with no significant differences between time points (AMOVA, *P* > 0.05; HOMOVA, *P* > 0.05). Linear discriminant analysis effect size method was performed and failed to detect any enriched taxa, between time points. Random forest algorithm analysis indicated homogeneity across time points.

**Conclusions:**

Although existing evidence from human fecal storage studies is contradictory, a recent study in companion animals agreed with the current study, demonstrating that maintenance of feline fecal samples at ambient temperature for up to 4 days has no effect on the bacterial membership and structure.

## Background

The human gastrointestinal (GI) microbial community (the ‘microbiota’) is extremely complex in composition, and very high in concentration, reaching 10^12^–10^14^ cell/g of intestinal content. The microbiota is dominated by bacteria, but also consists of Archaea, viruses, fungi and parasites. The bacterial concentration progresses from the stomach to the colon, with the highest concentration found in the colon [1–4]. Similar numbers are found in dogs and cats, although composition and bacterial species dominance are different from humans [5, 6]. Increased evidence exists in humans and companion animals for the health implications and clinical importance of the commensal or symbiotic relationship between the intestinal bacteria and their host. The intestinal microbiota plays a crucial role in the development of the host immune system, protection against pathogens, toxins and mutagens and utilization of excess nutrients or nutrients that are unavailable to the host [7]. The microbiota produces short chain fatty acids (SCFAs) that serve as energy sources to the colonocytes, produces vitamins, and aids in mineral absorption and intestinal integrity, along with a myriad of other effects, many of which remain to be properly defined [5, 8, 9].

Any deviation from the ‘normal’ microbiota is referred to as intestinal dysbiosis [5, 10]. In humans, animal models and companion animals, dysbiosis can be associated with a range of disease states, particularly inflammation [11–14]. Alterations of the normal gut microbiota balance [15, 16], due to inherent, environmental or immunological factors can be involved in the pathogenesis of intestinal inflammatory diseases [11, 17–19], or with other organ-related diseases, such as diabetes mellitus, obesity or asthma [2, 20–22].

In order to characterize the human or animal microbiota, a variety of methods can be used. In the past, the use of bacterial cultivation techniques dominated, but results are limited because of the inability to grow a large percentage of the microbiota using standard culture methods [23, 24]. Recently developed high-throughput techniques, such as Illumina MiSeq, are capable of quick massive parallel sequencing, providing more into-depth understanding of human and companion animal microbiome, and are considered as the preferred analytical method nowadays [25].

Aside to the effects of DNA extraction methods [26] or molecular tools, fecal sampling technique and sample storage conditions can potentially influence phylogenetic identification. The effects of different sampling and storage methods on the fecal bacterial population of healthy and diseased human subjects were examined [27]. Fecal samples were aliquoted within 10 min of defecation and stored at different temperatures: -80 °C, at -20 °C for a week, at +4 °C for 24 h and at ambient temperature for 24 h. No significant differences were found in the number of operational taxonomic units (OTUs), diversity or richness between the different storage temperatures [27]. This study agreed with the results of previous studies [28], but is in contradiction with others, that reported a mild gradual difference in the fecal bacterial composition when samples were stored at ambient temperature for 24 h compared to the composition assessed around defecation [29, 30]. Studies evaluating the impact of storage conditions on fecal microbiota in companion animals are scarce. One study in dogs and cats identified limited change in the microbiota from short term (<2 week) refrigeration [31]. However, the impact of room temperature storage was not assessed. This is an important aspect to understand for field studies, particularly of species such as cats. Fecal collections for microbiota-related studies in client-owned cats are often challenging, as significant time may pass between defecation, sample collection, and submission for analyses. During this time the sample may be left at room temperature (e.g. overnight in the litter box) before proper storage. The objective of this study was to determine the effect of ambient temperature exposure on the feline fecal microbiota.

## Methods

### Sample collection

Fresh fecal samples were collected from 12 healthy cats located at a cat boarding facility in Guelph, Ontario from June to July 2016. The subjects were determined to be healthy based on information provided to the facility manager by cat owners prior to boarding. Cats were observed closely by facility personnel and fecal samples were collected within 15 min of defecation. Samples were maintained unrefrigerated until arrival at the laboratory. Upon arrival, samples were weighed,^1^ manually homogenized and aliquoted into 200 mg samples. One aliquot was frozen at −80 °C within 1 hour of defecation (time point (T) 0). The remaining aliquots were kept at ambient temperature (20 to 23 °C) in a biosafety cabinet.^2^ Aliquots were frozen at −80 °C at the following time points: 6, 12, 24, 36, 48, 72, 96 h. Downstream processing was then performed on samples that had all undergone the same freeze-thaw cycle and were processed as a batch.

### DNA extraction

For DNA extraction, a commercial stool extraction kit^3^ was used according to the manufacturer’s instructions. The DNA was collected in 1.5 ml micro-centrifuge tubes, and stored at −80 °C until further analysis.

### Polymerase chain reaction (PCR)

The quantity of extracted DNA was assessed using a spectrophotometer,^4^ with readings ranging from 33 to 661 ng/ml. To prepare the 16S rRNA gene amplicons for the Illumina MiSeq system,^5^ all DNA samples were diluted (if needed) to a range of 30 to 100 ng/ml. The V4 region of the 16S rRNA gene was amplified using the forward primer S-D-Bact-0564-a-S-15 (5′-AYTGGGYDTAAAGNG-3′), reverse primer S-D-Bact-0785-b-A-18 (5′-TACNVGGGTATCTAATCC-3′), KAPA HiFi ReadyMix,^6^ and PCR grade water. The PCR cycles were conducted in a thermal cycler.^7^ The purified PCR products were evaluated with 1.5% agarose gel for gel electrophoresis and DNA was measured using spectrophotometry.

### DNA sequencing

Using an Illumina MiSeq system, the samples were amplified by bridge amplification and sequenced with terminator nucleotides [32]. At least 100,000 reads/sample with sequences of approximately 500 bp in length from 2 × 250 paired end reads were obtained [32].

### Bioinformatics analyses and statistics

Mothur v1.36.1 was used for bioinformatics analyses, as well as some of the statistical analyses [33, 34]. Additional statistical analyses were performed using JMP 13.0.^8^ Paired end reads were assembled and filtered to remove sequences greater than 250 base pairs (bp) in length. Sequences with any ambiguous base calls or runs of homopolymers greater than 8 bp were removed. Sequences were aligned to the Silva16S rRNA reference database [35], and those that did not align with the correct region were removed. In addition, chimeras were identified using uchime [36] and removed. Sequences were classified using the RDP classifier (v14) [37], and those taxonomic assignments were used to create OTUs using a closed (database-dependent) OTU picking approach. Archeae were removed. Subsampling was performed based on the smallest number of sequences from a sample, to standardize sequence number used for analysis [38].

Alpha-diversity indexes (Shannon diversity [39], Simpson diversity [40] and Chao1 [41]) were calculated to assess evenness, diversity and richness, respectively, and compared between time points using a nonparametric multiple comparisons test (Wilcoxon Each Pair). Relative abundances were calculated for the different taxonomic levels, at each time point. Differences in relative abundance of taxa accounting for ≥1% of sequences within phyla and ≥0.1% within genera were evaluated using nonparametric multiple comparison test (Wilcoxon Each Pair), with *p*-values adjusted using the Benjamini-Hochberg correction to control for the false discovery rate [42]. Beta-diversity was assessed, using the classical Jaccard index [43] and Yue & Clayton index of dissimilarity [44] to examine community membership and population structure, respectively. For visualisation of differences in membership and structure between cats and time points, dendrograms were created, and significance of clustering according to time point was determined using parsimony and unifrac unweighted [45]. Beta-diversity was visualized using principal coordinate analyses (PCoA) and further assessed using analysis of molecular variance (AMOVA) and homogeneity of molecular variance (HOMOVA). Linear discriminatory analysis (LDA) effective size (LefSe) [46] was conducted as well for the identification of genomic features between time points. Random forest algorithm analysis was used to assess the ability to predict group classification, by cat or by time point [47].

## Results

Ninety-three samples were processed from the 12 cats. Three aliquots (two at T96, and one at T72) were not obtained due to small fecal samples size from two cats. A total of 9,118,609 sequences passed all filters, with a median of 93,909 sequences per sample, and a range of 50,315 to 207,127 sequences per sample. A random subsample of 50,315 sequences per sample was used to normalize samples for analysis.

**Fig. 1:**
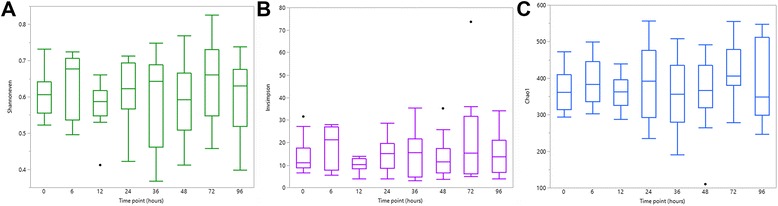
Comparison of fecal bacterial population evenness (**a**), diversity (**b**) and richness (**c**) in 12 healthy cats between time points 0, 6, 12, 24, 36, 48, 72 and 96 h

**Fig. 2:**
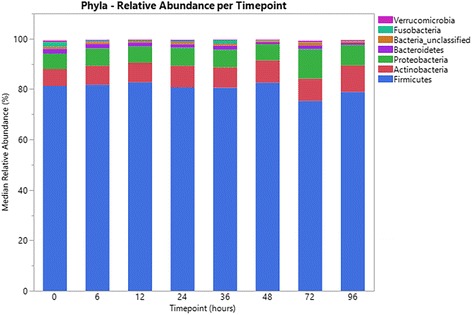
Comparison of median relative abundances of predominant phyla originating from fecal samples of 12 healthy cats, between time points 0, 6, 12, 24, 36, 48, 72 and 96 h

**Fig. 3:**
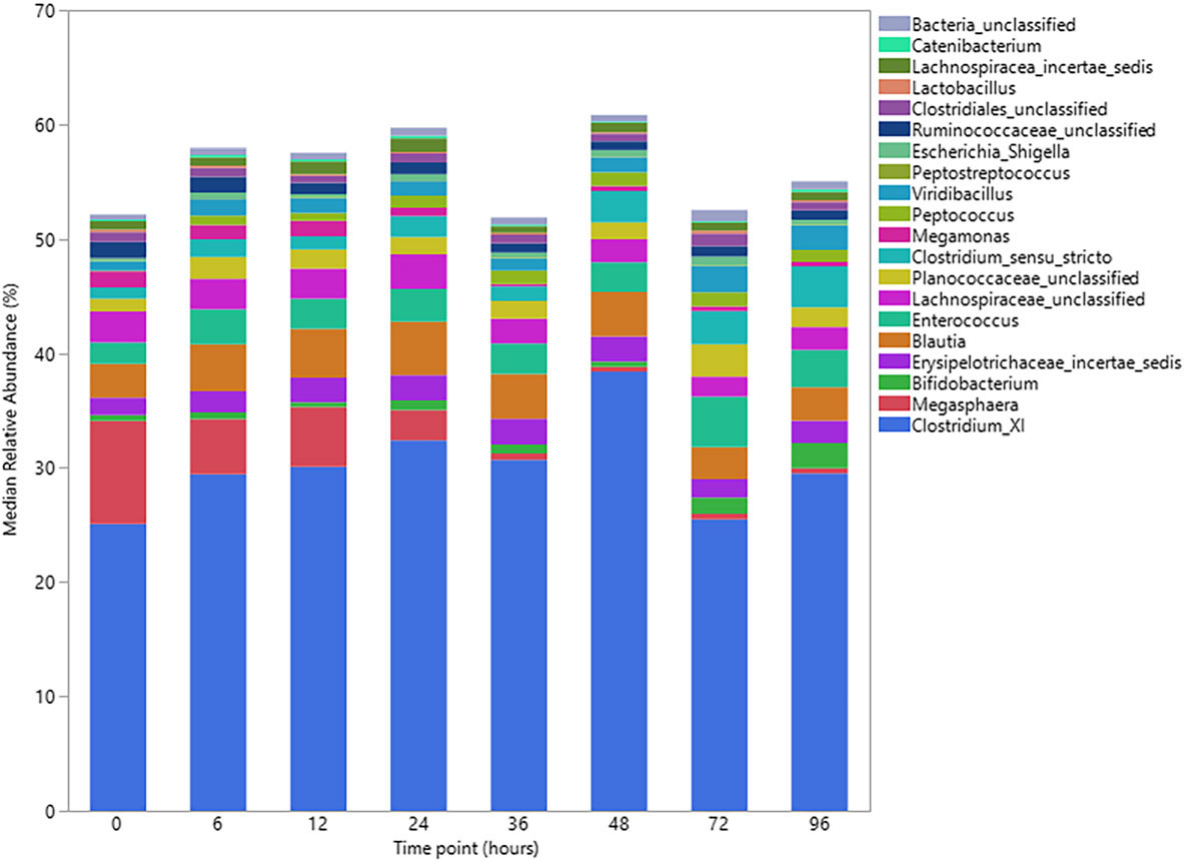
Comparison of median relative abundances of predominant genera originating from fecal samples of 12 healthy cats, between time points 0, 6, 12, 24, 36, 48, 72 and 96 h

There were no significant differences in evenness, diversity and richness between the different time points (all *P* > 0.05) (Fig. 1). Median bacterial relative abundance accounting for ≥1% of phyla and ≥0.1% of genera, are presented in Figs. 2 and 3 respectively. No significant differences in relative abundances were noted at any taxonomic level (all adjusted *P* > 0.05). There was a numerical decrease in *Megasphaera* (Fig. 3); however, this was not statistically significant (unadjusted *P* = 0.06, adjusted *P* = 0.81).

**Fig. 4:**
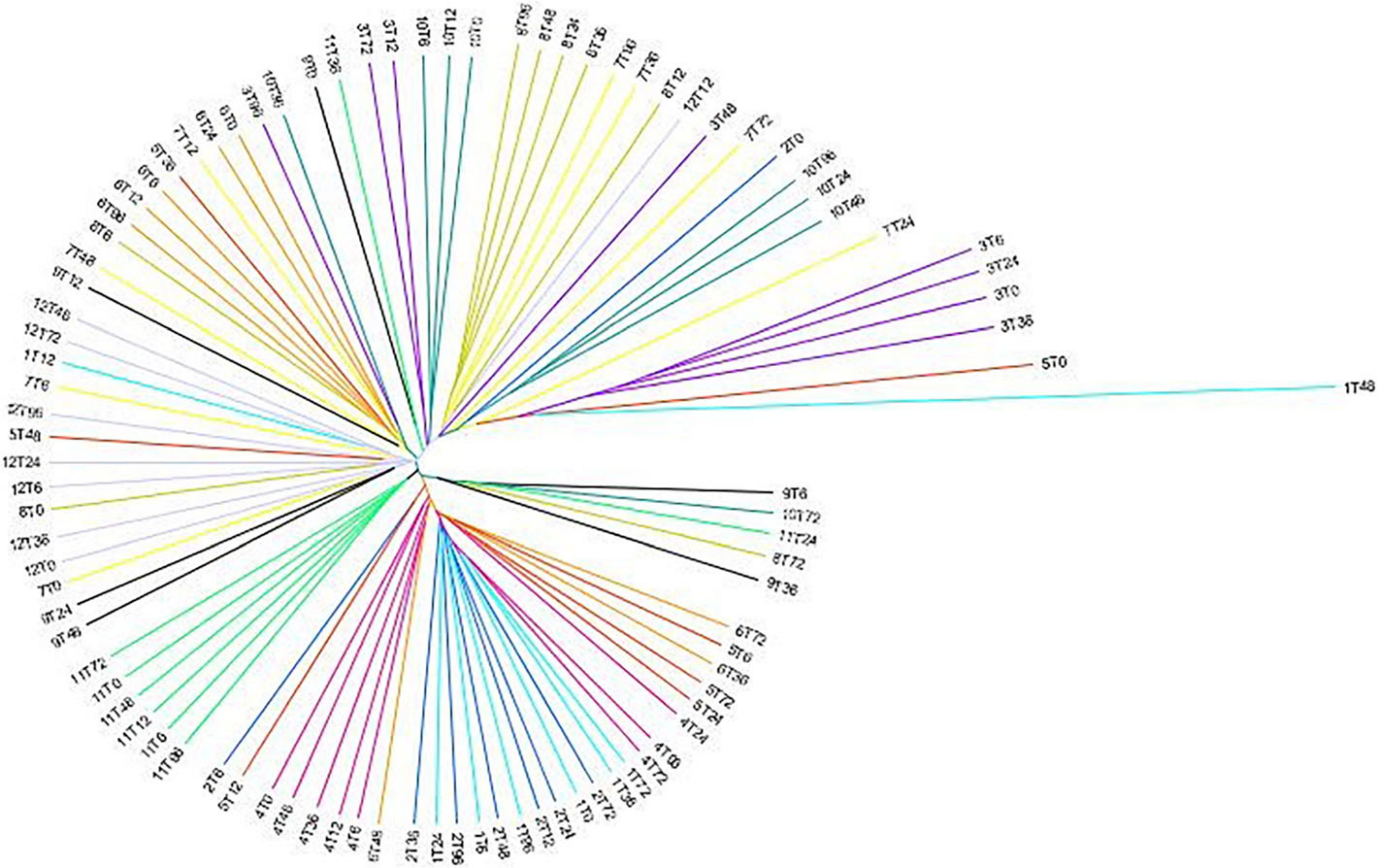
Dendrogram of the Classical Jaccard index representing the community membership of the fecal microbiota in 12 healthy cats, compared between time points 0, 6, 12, 24, 36, 48, 72 and 96 h. Each cat is represented with a different colour

**Fig. 5:**
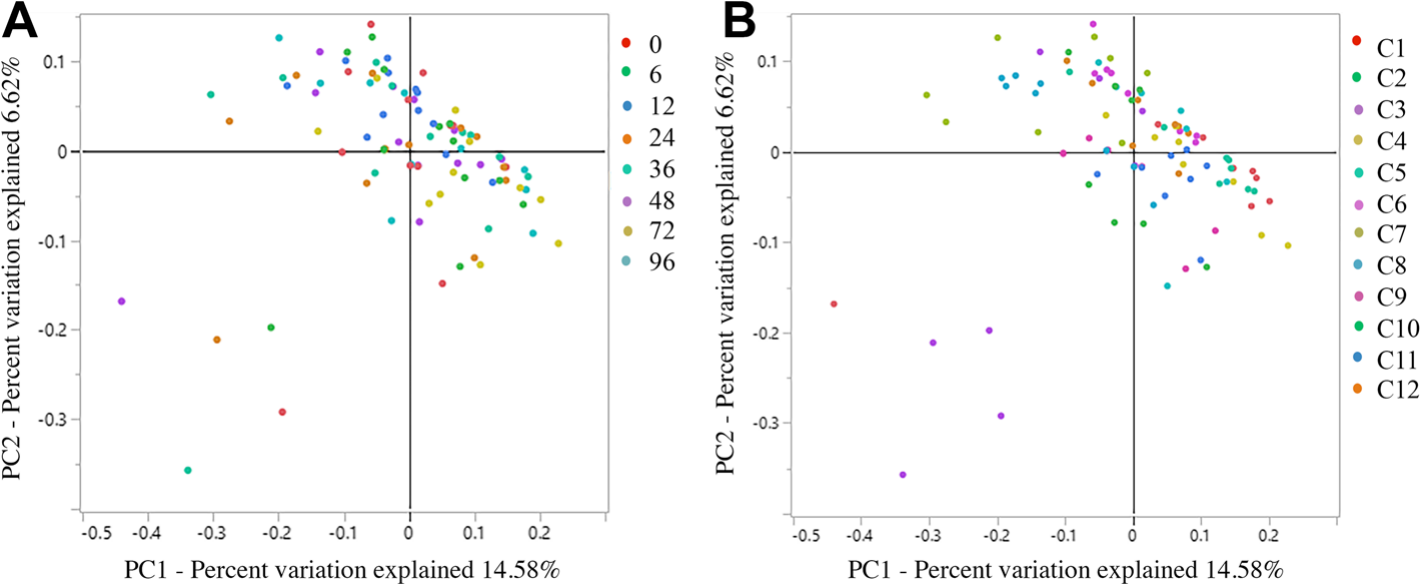
Two dimensional principal coordinate analysis of population membership of the fecal microbiota of 12 healthy cats, assessed at time points 0, 6, 12, 24, 36, 48, 72 and 96 h. (**a**) By time point; (**b**) By cat

No differences in community membership (Classical Jaccard index – unifrac *P* = 0.74; parsimony *P* > 0.05 for all comparisons) or population structure (Yue & Clayton – unifrac *P* = 0.83; parsimony *P* > 0.05 for all comparisons) were observed. Clustering by cat, but not time point, was apparent on the dendrograms for both community membership and structure (Fig. 4). Principal coordinate analyses indicated that fecal microbiota mainly clustered by cat, with no significant differences in community membership (Fig. 5a and b) or structure between time points (AMOVA *P* > 0.05 and HOMOVA *P* > 0.05 for all comparisons).

Linear discriminant analysis (LDA) effect size (LEfSe) analysis failed to identify enriched taxa between time points. Random forest algorithm analysis was performed both to evaluate the ability to differentiate between time points and between cats. When random forest was analysed according to time point, 92% error rate was established, indicating homogeneity across time points, and a very poor ability to separate samples into their appropriate groups. However, when “cat” was analysed as the group, the error rate was only 15%, indicating a much stronger ability to assign samples to the appropriate cat.

## Discussion

The results from the study imply that there are no significant changes in feline fecal microbiota evenness, diversity and richness, as well as community membership and structure over 96 h, while fecal samples are kept at ambient temperature.

Microbiota assessment in feline fecal samples can provide important insight. However, multiple variables can impact the results. It is important to understand potential external influences or biases that might affect the ability to properly define the microbiota and detect true biological differences. The potential impact of sample storage is one potential concern, particularly in studies that involve collection of samples from the community and inherent delays until processing. Understanding the potential influences of sample storage is important for proper design and interpretation of studies.

A variety of ecological indices can be used to assess microbial biodiversity. Despite the use of a range of methods, including assessment of relative abundances and alpha and beta diversity indices, no significant impact of storage on the fecal microbiota was identified. In addition to statistical analyses, clear numerical, non-statistically significant, differences were evident. While care must be taken when considering any non-significant results, these results should not be completely dismissed as statistical differences can be clouded by the degree of inter-sample variation and power limitations. The most readily apparent numerical difference was a decrease in the relative abundance over time of *Megasphaera,* a genus of relatively fastidious anaerobes [48]. Therefore, the trend for decreased *Megasphaera* abundance over time could correspond to air exposure during storage, something that might have been accentuated by manual homogenization during preparation of fecal samples. However, since the methods used in this study do not depend on viable microorganisms, it is unclear whether poor aerotolerance can accurately explain these results. *Megasphaera* was shown to play a dominant role in bacterial composition of the feline GI tract [49]. It is associated with ruminal fermentation of lactate into short-chain fatty acids (SCFA), and is especially related to butyrate formation [50]. Due to its fermentation capacity, the bacterium was shown to have beneficial effects on the GI health of some monogastric mammals [51, 52]. However, further research is required for the understanding of the underlined role of *Megasphaera* in the cat, an obligate carnivore [49], especially when increased protein content may promote a steep decrease in *Megasphaera* abundance, as well as jeopardise the GI microbiota health [53].

While the extent of studies investigating the impact of storage on feline fecal microbiota has been limited, the results of this study are similar to other studies that have shown limited or no impact of short-term storage. One study investigated the effect of storage at 4 °C on fecal samples from seven dogs and ten cats, after 0, 3, 7 and 14 days [31]. Only a couple of significant changes were observed in the feline fecal microbiota, such as a decrease in the relative abundance of Erysipelotrichaceae *incertae sedis* after 7 days, or enrichment with *Psychrobacter* and *Arthrobacter* (Proteobacteria and Actinobacteria respectively) after 14 days [31]. In comparison to this refrigeration study, it would be expected that changes would occur prior to 7 days of storage at ambient temperature, which was not observed in the current study. A human study involving fecal samples from four healthy children, kept at room temperature for 12, 24, 48 and 72 h, reported a minor change in community structure over time [29]. A small but significant increase in diversity was identified after 12 h along with a decrease in strains of *Ruminococcus* and *Faecalibacterium* [29]. Corresponding changes were not noted in the present study, with possible explanations being differences in sample preparation as well as inter-species variation in fecal microbiota composition. In contrast to the study by Roesch et al. (2009), non-sterile containers were used for sample preservation in the current study and fecal samples were homogenised, as performed similarly by Weese et al. (2014) [29, 31]. Homogenization of stool assists in standardization of aliquots [4, 31, 54]. However, some researchers opt not to homogenise samples as it also causes increased oxygen exposure, which can influence the microbiota membership and structure over time [27]. Additional methodological parameters that can contribute to differences in sample membership and population structure are freeze-thaw cycles [55], differences in extraction methods [56] as well as sequencing methods. Regardless, while there have been variable results from studies in different species using different storage conditions, changes noted over the short timeframe of this study have been mild or absent, consistent with these data.

In general, changes in membership or population structure would correlate to either degradation of microbial DNA or bacterial growth. Dietary ingredients may include microbial metabolic inhibitors. Most feline diets are composed of both animal products and plants ingredients. Plant ingredients contain chlorophyll metabolites such as pheophorbide *a* and pyropheophorbide *a* [57], which inhibit bacterial efflux pumps [58]. The inhibitory effect of these metabolites could be one potential explanation for the lack of bacterial growth in the current study. Nonetheless, the dietary history of the cats was unavailable and further tests were not conducted to confirm this assumption.

Fecal samples in the current study were stored only for four days. This should be sufficient time in field studies to transport samples to better storage conditions, such as refrigeration or freezing. The sample size was relatively small, but it was based on a recent storage study in companion animals [31], as well as on human storage studies, that used a similar smaller sample size. Nonetheless, it is possible that with a larger sample size numerical trends in microbiota composition would become significant. Since very little research exists on the effects of storage on fecal samples in general, and even more so in cats, more research is warranted prior to solid conclusions in regards to short-term fecal storage recommendations.

## Conclusions

This study demonstrate that several-day-storage of healthy cats’ feces at ambient temperature, has no effect on microbial biodiversity. Although sample freezing at −80 °C is recommended for long term storage, the current study suggests that short term storage, up to 4 days, at ambient temperatures can be appropriate, especially when field studies are performed.

## Footnotes

1 Denver MAXX, Denver Instrument, Bohemia, New York, USA

2 Class II, Type A2 Biosafety 283 Cabinet, Thermo Fischer Scientific, Waltham, Massachusetts, USA

3 E.Z.N.A. Stool DNA Kit, 284 Omega Bio-Tek Inc., Doraville, Georgia, USA

4 NanoDrop 1000 Spectrophotometer, Nano 285 Drop Technologies Inc. (Thermo Fisher Scientific), Waltham, Massachusetts, USA

5 Illumina, San Diego, California, USA

6 Kapa Biosystems, Wilmington, Massachusetts, USA

7 Mastercycler Pro, Eppendorf Canada Ltd., Mississauga, Ontario, Canada;

8 SAS Campus Drive, Cary, North Carolina, USA
